# Esthetic preferences of orthodontists, general dentists, and laypersons for Indian facial profiles: A cross-sectional study

**DOI:** 10.12688/f1000research.138742.2

**Published:** 2024-09-30

**Authors:** Harshini Reddy, Ritesh Singla, Nishu Singla, Madhumitha Natarajan, Deepak Kumar Singhal

**Affiliations:** 1 Department of Orthodontics & Dentofacial Orthopaedics, Manipal College of Dental Sciences, Manipal, Manipal Academy of Higher Education, Manipal, Karnataka, 576104, India; 2 Department of Public Health Dentistry, Manipal College of Dental Sciences, Manipal, Manipal Academy of Higher Education, Manipal, Karnataka, 576104, India

**Keywords:** Indian facial profiles, esthetic, perceptions, orthodontists, general dentists, layperson

## Abstract

**Background:** Disparity in the esthetic perceptions between a patient and clinician could result in patient dissatisfaction with orthodontic treatment outcomes. The aim of this study was to compare the perceptions of a group of orthodontists, general dentists, and laypersons about the attractiveness of Indian facial profiles.

**Methods:** In this study, a male and a female participants’ photographs and lateral cephalograms were digitally manipulated by inserting them into Dolphin software; we considered four soft tissue parameters at a nasolabial angle, upper lip E-line, lower lip E-line, and pg-pg’, so that 20 profiles were created for each model. A visual analog scale (VAS) along with a question about surgical correction opinion was given to 18 orthodontists, 18 general dentists, and 18 laypersons to score (1-5) from least to most attractive. Spearman’s rank correlation was computed to assess correlation, as well as ANOVA, followed by *post hoc* Tukey analysis to compare the mean scores, and Chi-square test to determine the opinion about surgical treatment.

**Results:** There was an overall weak and negative correlation between the three groups, indicating that orthodontists attributed lower pleasantness scores to almost all the altered female and male facial profiles. Additionally, statistically significantly lower mean scores were attributed by orthodontists to many females and few male facial profiles. More orthodontists identified the need for surgical correction for a few severely distorted profiles but there was a statistically non-significant difference among the groups for most of the profiles.

**Conclusions:** It was concluded that participants in the three groups had diverse concepts of facial attractiveness in all the parameters considered. Compared to general dentists and laypersons, orthodontists were much more precise, firmer, and meticulous in identifying a favorable or good-looking profile.

## Introduction

Facial aesthetics are associated with how others perceive an individual. ^1^ The perception of an individual as being beautiful greatly impacts how they represent themselves to others, and therefore esthetics are not absolute, but highly subjective and variable. ^2^ Beauty is said to be “in the eyes of the beholder”. ^3^ Each person has a different intellectual wisdom about beauty and its perception varies accordingly. Most of it is influenced by principles of one’s self-perception. The perception of one’s disfigurement, flaws, or imperfections may be beautiful to another. Women’s feet in China were bound to make them small which was perceived more attractive. ^4^ Midline diastema was considered aesthetic among Arabs. ^5^

Facial esthetics have always been an integral part of the standards and practice of orthodontics. Even though standard occlusion remains a chief functional goal, achieving proper esthetic outcomes remains essential for the satisfaction of the patients. Individuals with an ideal dentofacial appearance are considered more good-looking socially than those with an unaesthetic dental appearance and often have low self-confidence. ^6^ Holdaway suggested that identifying soft tissue traits that contribute to or reduce physical attractiveness stereotypes deeply rooted in society can enhance treatment objectives. ^7^ Orthodontists usually judge the characteristics of facial esthetics based on smile assessments of the patient, profile, and full face. Over the years, clinical perceptions of facial esthetics have progressively shifted to using quantifiable soft-tissue diagnostic valuations. Other orthodontic treatment methods to gain esthetic considerations – camouflage versus correction of jaw relationships, expansion versus extraction – have become important. ^7^

The treatment should be made to achieve a harmonious facial profile with an esthetically attractive smile and functional dental occlusion. When facial attractiveness and occlusion correction are combined, treatment planning becomes difficult. Achieving an ideal occlusion does not essentially mean that decent facial balance is achieved. To achieve accurate soft tissue effect to hard tissue changes, an orthodontist should concentrate on the growth and development of soft tissue traits. They should be aware of soft tissue changes produced by orthopedic or orthodontic treatment procedures. Hence, an orthodontist should conduct a thorough facial hard tissue and soft tissue examination so that orthodontic treatment can favorably affect the facial features. ^8^

In pursuing the ideal treatment goal, aesthetics has become crucial. Therefore, it is important to consider both the aesthetic perspectives of patients and clinicians regarding facial attractiveness. The difference in perception between the clinician and patient could result in patient dissatisfaction with treatment outcomes, as the perception of esthetics may vary between an orthodontist and a layperson. Thus, this study aimed to compare the perceptions of orthodontists, general dentists, and laypersons about the attractiveness of Indian facial profiles. In addition, it was also determined whether surgical treatment was needed for the profiles.

## Methods

A cross-sectional study was conducted considering three groups of study participants: orthodontists, general dentists, and laypersons (who were well-educated and not from a medical background) between the ages of 18 to 50 years. The total sample size was 54 with 18 subjects in each group. Ethical clearance was obtained from the Institutional Ethical Committee of Kasturba Medical College and Kasturba Hospital (IEC approval number 790/2018) for the conduct of the study. The study participants were informed about the study through a participant information sheet following which written informed consent was obtained. A visual analog scale (VAS) and a question about surgical correction opinion were given to 18 orthodontists, 18 general dentists, and 18 laypersons to score (1-5) from least to most attractive. The time taken to answer the questionnaire was approximately 10 min.

The photographs and lateral cephalograms of a 22-year-old male and a 21-year-old female were taken as study models. The standards for choosing these models comprised a well-balanced face with normal eyes, nose, and lips; class I skeletal and dental relationship; well-aligned arches; Z angle 71° - 89°. The exclusion criteria for selecting study models were class II and class III malocclusions, congenital deformities, and facial anomalies. Four soft tissue parameters were considered: nasolabial angle, upper lip E-line, lower lip E-line, and pg-pg’ (hard tissue pogonion to soft tissue pogonion). Most of the measurements of the role models were within the normal ranges. ^9^^,^^10^

**Figure 1.**
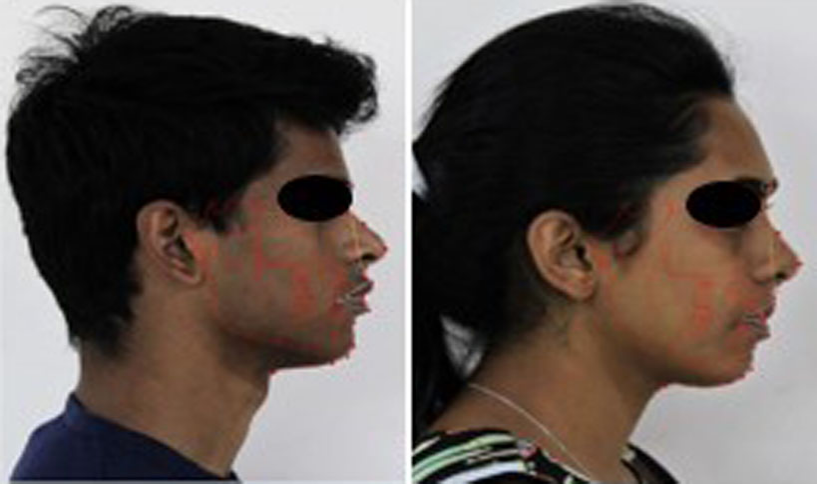
Lateral cephalograms were linked to their respective profile images by inserting them in the software.

Lateral cephalograms and standard high-resolution color profile photographs were taken with a white background and good brightness. Tracing the lateral cephalograms was done using Dolphin software (Dolphin Imaging 11.95 Premium Software). Lateral cephalograms were linked to their respective profile images by inserting them in the software, and nasolabial angles, E-Lines, and pg-pg’ were defined in the original pictures ( Figure 1). A total of 12 anatomic landmarks (eight soft tissue, four hard tissue) were recognized. The hard tissue landmarks were porion (po), sella (s), orbitale (or), and pogonion (pg). The soft tissue landmarks were pronasale (Pn), subnasale (Sn), superior labial sulcus (SLS), labrale superius (Ls), labrale inferius (Li), inferior labial sulcus (ILS), and soft tissue pogonion (pg’) and soft tissue menton (Mn’).

The pictures were digitally manipulated using the software by altering the lower component (labial part) of the nasolabial angle by 2° increments (increase and decrease); thus, a set of five profiles (one basic profile, two profiles with increase of nasolabial angle, two profiles with decrease of nasolabial angle) were created for each subject. The images were digitally manipulated for lower lip to E-Line and upper lip to E-Line by increasing 1 mm for two profiles and decreasing 1 mm for two profiles, and the last profile remains as the basic profile.

**Figure 2.**
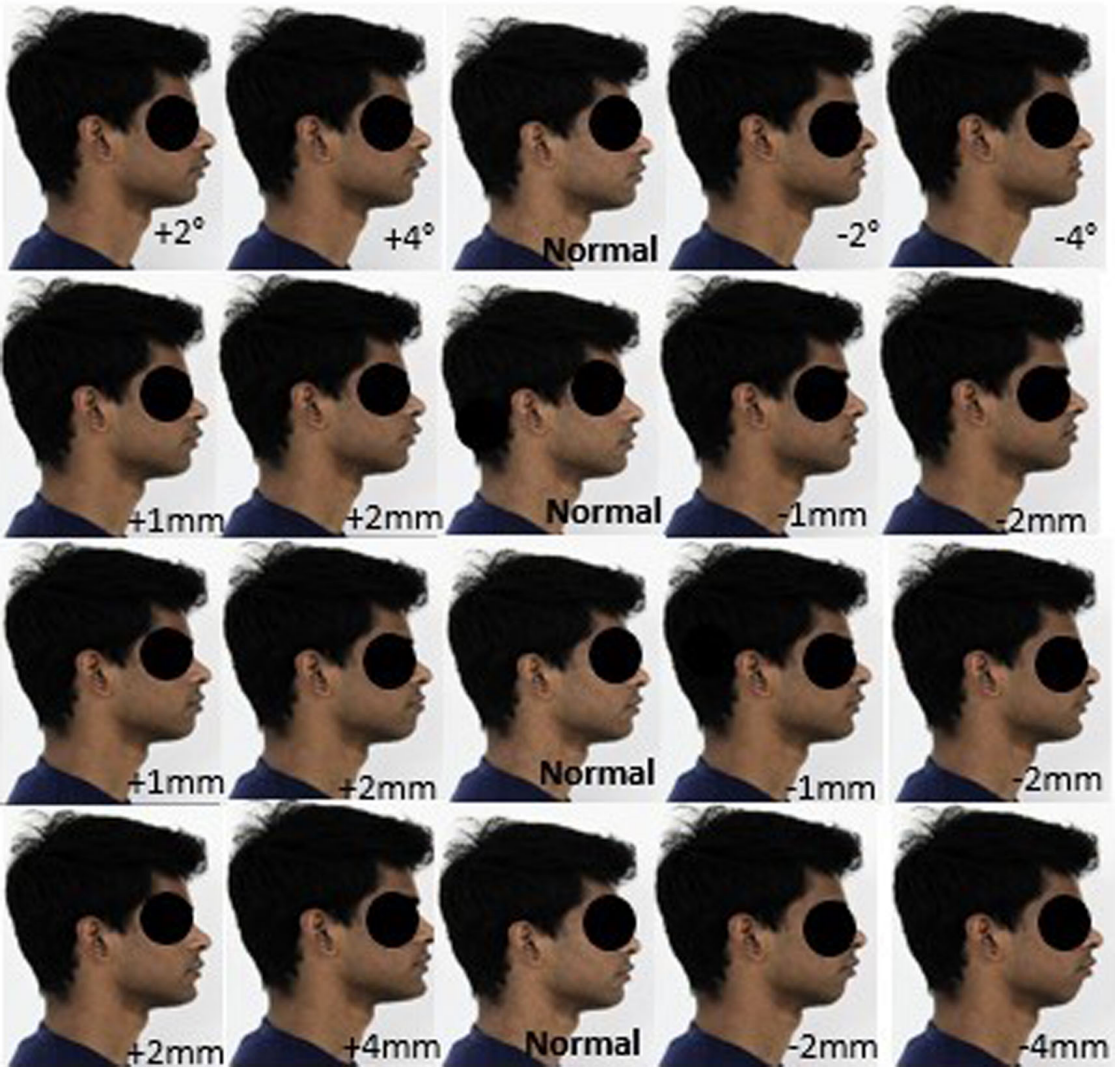
A set of 20 constructed profile images of male by altering nasolabial angle in first row, Upper lip to E-line in second row, Lower lip to E-line in third row and Hard tissue pogonion to soft tissue pogonion in fourth row.

**Figure 3.**
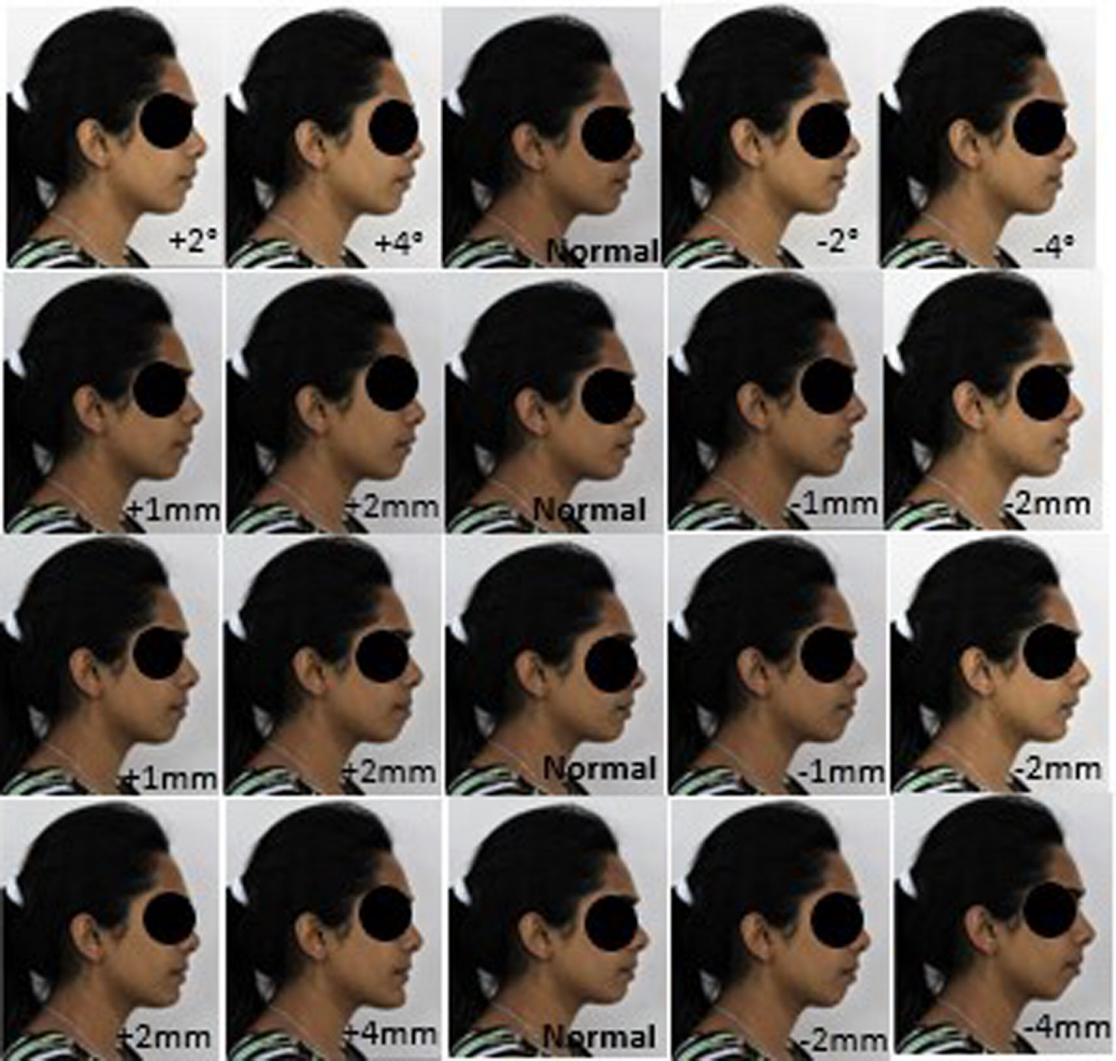
A set of 20 constructed profile images of female by altering nasolabial angle in first row, Upper lip to E-line in second row, Lower lip to E-line in third row and Hard tissue pogonion to soft tissue pogonion in fourth row.

The images were also digitally manipulated for pg-pg’ by increasing 2 mm for two profiles and by decreasing 2 mm for two profiles and the last profile remains as the basic profile. Thus, a total of 20 facial profiles were created for each model ( Figures 2 and 3): five profiles for nasolabial angle; five profiles for the upper lip E-line, five profiles for lower lip E-line; and five profiles for pg-pg’.

Once the profiles were reconstructed, the margins of the photos were blended out with Paint Shop Pro (version 7.0) to preserve the natural appearance of the pictures. Finally, the printed copies of the pictures were taken and randomly arranged in an album with no distinct order for both female and male subjects. As consented, no masking of the face was done during the study process as it involves the assessment of facial attractiveness. The study participants were assigned to score (1-5) for each image through a Visual Analogue Scale (VAS) from least to most attractive. The participants were also asked a yes/no question to answer whether they thought the profile required any surgery to improve their facial appearance.

### Statistical data analysis

Data was entered into Microsoft Excel and evaluated using statistical software SPSS version 20. Spearman’s rank correlation was computed to assess correlation and ANOVA, followed by *post hoc* Tukey analysis to compare the mean scores and the Chi-square test to determine the opinion about surgical treatment. The groups were ranked for Spearman’s rank correlation based on their increasing levels of orthodontic knowledge, such as laypersons followed by general dentists and orthodontists. The Spearman’s rank correlation coefficient ( *R _s_*) value lies between 1.0 and -1.0 (a perfect positive to negative correlation). The strength of a correlation depends on the value of the coefficient. A *R _s_* between 0.00 - 0.19 implies a very weak, 0.20 - 0.39 a weak, 0.40 - 0.69 a moderate, 0.70-0.89 a strong, and 0.90-1.00 a very strong correlation. Also, a R _s_ of 0 indicates no association between ranks. The 5% probability level (p ≤ 0.05) implied a statistically significant difference.

## Results

According to the results of the Spearman’s rank correlation test, there was an overall weak, negative correlation between the three groups for the VAS scores for most of the female and male profiles. A negative correlation indicates that the orthodontist’s group of the highest rank gave the lowest pleasantness scores to almost all the altered female and male facial profiles on a VAS of 1-5 compared to other groups. A statistically significant but weak negative correlation was seen between the three study groups for female profiles with Nasolabial angle (+4°), Nasolabial angle (-4°), Upper lip to E-line (+2 mm), Upper lip to E- line (-1 mm), Lower lip to E-line (normal), Lower lip to E-line (-2 mm), pg-pg’ (-2 mm) and pg- pg’ (-4 mm); a moderate negative correlation for female profiles with Upper lip to E-line (-2 mm) and Lower lip to E-line (-1 mm). There was no correlation between the three groups for the profile Upper lip to E-line (normal) and a positive but very weak correlation for profiles with Nasolabial angle (normal) and Nasolabial angle (-2°). In contrast, the remaining female profiles showed statistically non-significant weak negative correlation as shown in Table 1. For male profiles, it was found that there was a statistically significant negative weak correlation between the three study groups for profiles with Nasolabial angle (+2°) and Nasolabial angle (+4°). In contrast, all remaining male profiles showed a statistically non-significant and very weak negative correlation.

**Table 1.** Results of Spearman’s Rank Correlation between three study groups.

	Spearman’s Correlation for female facial profiles	P value	Spearman’s Correlation for male facial profiles	P value
Nasolabial angle (+2°)	-0.242	0.08	-0.305	**0.03** *
Nasolabial angle (+4°)	-0.284	**0.04** *	-0.329	**0.02** *
Nasolabial angle (normal)	0.09	0.52	-0.017	0.903
Nasolabial angle (-2°)	0.05	0.72	-0.080	0.566
Nasolabial angle (-4°)	-0.332	**0.02** *	-0.234	0.089
Upper lip to E-line (+1 mm)	-0.235	0.09	-0.264	0.073
Upper lip to E-line (+2 mm)	-0.283	**0.04** *	-0.229	0.096
Upper lip to E-line (normal)	0.0	1	-0.062	0.656
Upper lip to E-line (-1 mm)	-0.294	**0.03** *	-0.146	0.293
Upper lip to E-line (-2 mm)	-0.412	**0.002** *	-0.230	0.094
Lower lip to E-line (+1 mm)	-0.248	0.07	-0.140	0.314
Lower lip to E-line (+2 mm)	-0.193	0.16	-0.127	0.361
Lower lip to E-line (normal)	-0.267	**0.05** *	-0.0 I	0.944
Lower lip to E-line (-1 mm)	-0.453	**0.001** *	-0.238	0.084
Lower lip to E-line (-2 mm)	-0.374	**0.005** *	-0.262	0.056
pg-pg’ (+2 mm)	-0.149	0.283	-0.154	0.266
pg-pg’ (+4 mm)	-0.199	0.149	-0.096	0.488
pg-pg’ (normal)	-0.073	0.598	-0.078	0.576
pg-pg’ (-2 mm)	-0.301	**0.03** *	-0.147	0.289
pg-pg’ (-4 mm)	-0.313	**0.02** *	-0.199	0.148

* P value ≤ 0.05 was considered a statistically significant difference.

The results of ANOVA presented in Table 2 show that the orthodontist group attributed lower mean scores to almost all the altered female and male facial profiles. However, statistically significant differences in mean scores were noted for female profiles with Nasolabial angle (+4°), Nasolabial angle (-4°), Upper lip to E-line (-1 mm), Upper lip to E- line (-2 mm), Lower lip to E-line (normal), Lower lip to E-line (-1 mm), Lower lip to E-line (-2 mm), pg-pg’ (+4 mm), pg-pg’ (normal) and pg-pg’ (-4 mm). *Post hoc* analysis showed that for female profiles with Lower lip to E-line (normal) and pg-pg’ (normal), orthodontists significantly differed from general dentists; for profiles with Nasolabial angle (+4°), Nasolabial angle (-4°), Upper lip to E-line (-1 mm), Upper lip to E-line (-2 mm), Lower lip to E-line (-1 mm), Lower lip to E-line (-2 mm) and pg-pg’ (-4 mm), orthodontists significantly differed from laypersons. In contrast, for male profiles, only profiles with Nasolabial angle (+2°), Nasolabial angle (+4°), and pg-pg’ (+4 mm) were statistically different in the responses between the three groups, and the *post hoc* analysis showed that for profiles with Nasolabial angle (+2°) and Nasolabial angle (+4°), orthodontists significantly differed from laypersons. Meanwhile, all remaining profiles of the male model showed statistically non-significant differences between the three groups ( Table 2).

**Table 2.** Results of ANOVA followed by post hoc Tukey analysis between three study groups.

	Groups	VAS scores for Female model (Mean ± SD)	P value	VAS scores for Male model (Mean ± SD)	P value
Nasolabial angle (+2°)	Layperson	3.56 ± 0.705	0.118	3.00 ± 0.686 ^**a**^	**0.024** *
	General Dentist	3.28 ± 0.461		3.06 ± 0.639 ^**a**^	
	Orthodontist	3.17 ± 0.514		2.50 ± 0.618 ^**b**^	
Nasolabial angle (+4°)	Layperson ^**b**^	3.44±0.616	**0.023** *	2.72 ± 0.826 ^**a**^	**0.032** *
	General Dentist ^**a**^	3.50 ± 0.618		2.67 ± 0.485 ^**ab**^	
	Orthodontist ^**ab**^	3.00 ± 0.485		2.22 ± 0.428 ^**b**^	
Nasolabial angle (normal)	Layperson	3.22 ± 0.808	0.209	3.56 ± 0.616	0.663
	General Dentist	3.6 1 ± 0.608		3.72 ± 0.575	
	Orthodontist	3.39 ± 0.502		3.56 ± 0.705	
Nasolabial angle (-2°)	Layperson	3.06 ± 0.938	0.918	3.11 ± 0.963	0.659
	General Dentist	3.17 ± 0.514		3.17 ± 0.618	
	Orthodontist	3.11 ± 0.900		2.94 ± 0.639	
Nasolabial angle (-4°)	Layperson ^**a**^	2.72 ± 1.227	**0.013** *	2.39 ± 0.916	0.115
	General Dentist ^**b**^	2.67 ± 0.686		2.00 ± 0.686	
	Orthodontist ^**b**^	1.94 ± 0.416		1.89 ± 0.583	
Upper lip to E-line (+1 mm)	Layperson	3.28 ± 0.669	0.146	3.22 ± 0.808	0.124
	General Dentist	2.94 ± 0.725		3.17 ± 0.618	
	Orthodontist	2.89 ± 0.471		2.78 ± 0.647	
Upper lip to E-line (+2 mm)	Layperson	3.22 ± 0.732	0.098	2.67 ± 0.767	0.141
	General Dentist	2.94 ± 0.725		2.56 ± 0.511	
	Orthodontist	2.72 ± 0.575		2.28 ± 0.461	
Upper lip to E-line (normal)	Layperson	3.67 ± 0.686	I	3.50 ± 0.514	0.172
	General Dentist	3.67 ± 0.485		3.78 ± 0.548	
	Orthodontist	3.67 ± 0.686		3.44 ± 0.616	
Upper lip to E-line (-1 mm)	Layperson ^**a**^	2.72 ± 0.958	**0.044** *	3.l l ± 0.758	0.287
	General Dentist ^**ab**^	2.44 ± 0.705		3.22 ± 0.428	
	Orthodontist ^**b**^	2.06 ± 0.639		2.89 ± 0.676	
Upper lip to E-line (-2 mm)	Layperson ^**a**^	2.50 ± 1.043	**0.006** *	2.56 ± 0.922	0.087
	General Dentist ^**ab**^	2.11 ± 0.471		2.56 ± 0.511	
	Orthodontist ^**b**^	1.67 ± 0.594		2.06 ± 0.802	
Lower lip to E-line (+1 mm)	Layperson	3.33 ± 0.686	0.165	3.39 ± 0.608	0.374
	General Dentist	3.11 ± 0.583		3.39 ± 0.502	
	Orthodontist	2.94 ± 0.539		3.17±0.514	
Lower lip to E-line (+2 mm)	Layperson	2.72 ± 0.958	0.198	2.72 ± 0.826	0.421
	General Dentist	2.56 ± 0.705		2.50 ± 0.618	
	Orthodontist	2.28 ± 0.461		2.44 ± 0.511	
Lower lip to E-line (normal)	Layperson ^**ab**^	3.89 ± 0.583	**0.033** *	3.78 ± 0.548	0.939
	General Dentist ^**b**^	3.44 ±0.511		3.83 ± 0.383	
	Orthodontist ^**a**^	3.50 ± 0.514		3.78 ± 0.647	
Lower lip to E-line (-1 mm)	Layperson ^**a**^	3.50 ± 0.707	**0.003** *	3.50 ± 0.707	0.068
	General Dentist ^**ab**^	3.22 ± 0.548		3.67 ± 0.594	
	Orthodontist ^**b**^	2.83 ± 0.383		3.17 ± 0.618	
Lower lip to E-line (-2 mm)	Layperson ^**a**^	3.28 ± 0.895	**0.011** *	3.00 ± 0.840	0.076
	General Dentist ^**b**^	3.11 ± 1.023		2.94 ± 0.539	
	Orthodontist ^**b**^	2.39 ± 0.778		2.50 ± 0.707	
pg-pg’ (+2 mm)	Layperson	3.78 ±0.647	0.564	3.78 ± 0.548	0.531
	General Dentist	3.67 ±0.594		3.72 ± 0.461	
	Orthodontist	3.56 ± 0.616		3.56 ± 0.784	
pg-pg’ (+4 mm)	Layperson ^**b**^	2.94 ±0.873	**0.032** *	3.17±0.786 ^**b**^	**0.016** *
	General Dentist ^**a**^	3.17 ± 0.618		3.6 I ± 0.502 ^**a**^	
	Orthodontist ^**ab**^	2.56±0.511		2.94 ± 0.725 ^**ab**^	
pg-pg’ (normal)	Layperson ^**ab**^	3.67 ±0.767	**0.026** *	3.61 ± 0.502	0.952
	General Dentist ^**b**^	3.11 ±0.471		3.6 I ± 0.608	
	Orthodontist ^**a**^	3.56 ± 0.616		3.56 ± 0.705	
pg-pg’ (-2 mm)	Layperson	2.94 ± 0.802	0.069	2.50 ± 0.857	0.379
	General Dentist	2.61 ± 0.778		2.44 ± 0.511	
	Orthodontist	2.39 ± 0.502		2.22 ± 0.428	
pg-pg’ (-4 mm)	Layperson ^**a**^	2.50 ± 1.043	**0.029** *	2.22 ± 1.114	0.164
	General Dentist ^**ab**^	2.00 ± 0.594		2.00 ± 0.485	
	Orthodontist ^**b**^	1.83 ± 0.514		1.72 ± 0.575	

* P value ≤ 0.05 is considered a statistically significant difference and for post hoc Tukey analysis groups with the same superscripted letter are not significantly different.

The results of the Chi-square test for female profiles with Lower lip to E-line (-2 mm), pg-pg’ (+4 mm), and pg-pg’ (-4 mm) showed that significantly more orthodontists preferred surgical correction as compared to other groups. Meanwhile, all remaining profiles showed statistically non-significant differences between the three groups. In contrast, for the male profile with Nasolabial angle (+4°), significantly more orthodontists preferred surgery as compared to other groups. The remaining profiles showed statistically non-significant differences between the three groups ( Table 3).

**Table 3.** Results of Chi-square Test for opinion for surgical treatment of three study groups.

	Groups	For female model		P value	For male model		P value
		Yes n (%)	No n (%)		Yes n (%)	No n (%)	
Nasolabial angle (+2°)	Orthodontist	1 (5.6%)	17 (94.4%)	1.00	5 (27.8%)	13 (72.2%)	0.424
	General Dentist	1 (5.6%)	17 (94.4%)		2 (11.1%)	16 (88.9%)	
	Layperson	1 (5.6%)	17 (94.4%)		3 (16.7%)	15 (83.3%)	
Nasolabial angle (+4°)	Orthodontist	4 (22.2%)	14 (77.8%)	0.317	10 (55.6%)	8 (44.4%)	**0.053** *
	General Dentist	2 (11.1%)	16 (88.9%)		3 (16.7%)	15 (83.3%)	
	Layperson	1 (5.6%)	17 (94.4%)		7 (38.9%)	11(61.1%)	
Nasolabial angle (normal)	Orthodontist	1 (5.6%)	17 (94.4%)	0.151	-	18 (100%)	-
	General Dentist	-	18 (100%)		-	18 (100%)	
	Layperson	3 (16.7%)	15 (83.3%)		-	18 (100%)	
Nasolabial angle (-2°)	Orthodontist	1 (5.6%)	17 (94.4%)	0.072	3 (16.7%)	15 (83.3 %)	0.381
	General Dentist	1 (5.6%)	17 (94.4%)		3 (16.7%)	15 (83.3%)	
	Layperson	5 (27.8 %)	13 (72.2%)		6 (33.3%)	12 (66.7%)	
Nasolabial angle (-4°)	Orthodontist	13(72.2%)	5 (27.8%)	0.064	13 (72.2 %)	5 (27.8%)	0.574
	General Dentist	6 (33.3%)	12 (66.7%)		1 l (61.1 %)	7 (38.9%)	
	Layperson	10 (55.6%)	8 (44.4%)		10 (55.6%)	8 (44.4%)	
Upper lip to E-line (+1 mm)	Orthodontist	3 (16.7%)	15 (83.3%)	0.570	2 (11.1%)	16 (88.9%)	0.105
	General Dentist	2 (11.1%)	16 (88.9%)		**-**	18 (100%)	
	Layperson	1 (5.6%)	17 (94.4%)		4 (22.2%)	14 (77.8%)	
Upper lip to E-line (+2 mm)	Orthodontist	5 (27.8%)	13 (72.2%)	0.148	10 (55.6%)	8 (44.4%)	0.407
	General Dentist	2 (11.1%)	16 (88.9%)		6 (33.3%)	12 (66.7%)	
	Layperson	1 (5.6%)	17 (94.4%)		8 (44.4%)	10 (55.6%)	
Upper lip to E-line (normal)	Orthodontist	-	18 (100%)	0.125	-	18 (100%)	0.361
	General Dentist	-	18 (100%)		-	18 (100%)	
	Layperson	2 (11.1%)	16 (88.9%)		1 (5.6%)	17 (94.4%)	
Upper lip to E-line (-1 mm)	Orthodontist	12 (66.7%)	6 (33.3%)	0.056	3 (16.7%)	15 (83.3%)	0.185
	General Dentist	5 (27.8%)	13 (72.2%)		-	18 (100%)	
	Layperson	10 (55.6%)	8 (44.4%)		3 (16.7%)	15 (83.3%)	
Upper lip to E-line (-2 mm)	Orthodontist	15 (83.3%)	3 (16.7%)	0.103	8 (44.4%)	10 (55.6%)	0.369
	General Dentist	9 (50%)	9 (50%)		5 (27.8%)	13 (72.2%)	
	Layperson	11(61.l %)	7 (38.9%)		9 (50%)	9 (50%)	
Lower lip to E-line (+1 mm)	Orthodontist	1 (5.6%)	17 (94.4%)	0.763	-	18 (100%)	0.125
	General Dentist	1 (5.6%)	17 (94.4%)		-	18 (100%)	
	Layperson	2 (11.l %)	16 (88.9%)		2 (11.1%)	16 (88.9%)	
Lower lip to E-line (+2 mm)	Orthodontist	7 (38.9%)	11 (61.1%)	0.393	4 (22.2%)	14 (77.8%)	0.436
	General Dentist	5 (27.8%)	13 (72.2%)		4 (22.2%)	14 (77.8%)	
	Layperson	9 (50%)	9 (50%)		7 (38.9%)	11 (61.1 %)	
Lower lip to E-line (normal)	Orthodontist	-	18 (100%)	-	-	18 (100%)	0.361
	General Dentist	-	18 (100%)		-	18 (100%)	
	Layperson	-	18 (100%)		1 (5.6%)	17 (94.4%)	
Lower lip to E-line (-1 mm)	Orthodontist	1 (5.6%)	17 (94.4%)	1.00	1 (5.6%)	17 (94.4%)	0.347
	General Dentist	1 (5.6%)	17 (94.4%)		-	18 (100%)	
	Layperson	1 (5.6%)	17 (94.4%)		2 (11.1%)	16 (88.9 %)	
Lower lip to E-line (-2 mm)	Orthodontist	11 (61.1%)	7 (38.9%)	**0.009** *	6 (33.3%)	12 (66.7 %)	0.368
	General Dentist	4 (22.2%)	14 (77.8%)		4 (22.2%)	14 (77.8%)	
	Layperson	3 (16.7%)	15 (83.3%)		8 (44.4%)	10 (55.6%)	
pg-pg’ (+2 mm)	Orthodontist	-	18 (100%)	-	-	18 (100%)	-
	General Dentist	-	18 (100%)		-	18 (100%)	
	Layperson	-	18 (100%)		-	18 (100%)	
pg-pg’ (+4 mm)	Orthodontist	7 (38.9%)	11 (61.1%)	**0.05** *	4 (22.2%)	14 (77.8%)	0.057
	General Dentist	1 (5.6%)	17 (94.4%)		1 (5.6%)	17 (94.4%)	
	Layperson	6 (33.3%)	12 (66.7%)		-	18 (100%)	
pg-pg’ (normal)	Orthodontist	-	18 (100%)	0.361	-	18 (100%)	-
	General Dentist	-	18 (100%)		-	18 (100%)	
	Layperson	1 (5.6%)	17 (94.4%)		-	18 (100%)	
pg-pg’ (-2 mm)	Orthodontist	9 (50%)	9 (50%)	0.105	10 (55.6%)	8 (44.4%)	0.743
	General Dentist	3 (16.7%)	15 (83.3%)		8 (44.4%)	10 (55.6%)	
	Layperson	6 (33.3 %)	12 (66.7%)		10 (55.6%)	8 (44.4%)	
pg-pg’ (-4 mm)	Orthodontist	17 (94.4%)	1 (5.6%)	**0.022** *	16 (88.9%)	2(11.1%)	0.301
	General Dentist	11(6 1.1%)	7 (38.9%)		16 (88.9%)	2(11.1%)	
	Layperson	10 (55.6%)	8 (44.4%)		13 (72.2 %)	S (27.8%)	

* P value ≤ 0.05 is considered a statistically significant difference.

## Discussion

In the present study, we evaluated the esthetic perceptions of different patient profiles from the standpoint of orthodontists, general dentists, and laypersons. The results showed an overall weak negative correlation between the three groups, which indicates that orthodontists attributed lower pleasantness scores to almost all the female and male facial profiles on the visual analog scale. Further, the results of ANOVA also showed that Orthodontists significantly attributed lower pleasantness scores to many females and few male facial profiles. Correspondingly, a similar study done by Volpato GM *et al.* reported that patients and lay people assigned higher pleasantness scores than orthodontists, with statistically significant differences for all evaluations. ^11^ Lines *et al.* and Imani *et al.* in their studies comparing judgments by orthodontists, general surgeons, other dental professionals, and laypersons, stated that even though the orthodontists’ group were more precise in their judgments when compared to oral surgeons, but their opinions still differed greatly from those of laypersons and other dental professionals. ^12^^,^^13^ In addition, as noticed in the present study, Romani *et al.* and Burcal *et al.*, specified that irrespective of the educational level the facial profiles of the female patients were judged more carefully. ^14^^,^^15^

In facial profiles with increased nasolabial angle by +4° and decreased nasolabial angle by -4°, orthodontists gave lower scores than the other two study groups for both female and male profiles. For a female profile where the lower component of the nasolabial angle was decreased by 2 degrees, a weak positive correlation or a fair agreement was seen between all three groups, as most of them agreed it to be an attractive profile. According to Farhad B. Naini *et al.,* ^16^ an upper lip inclination of 79°-85° is regarded as ideal, while a range of 73°-88° is considered acceptable. Angles lower than 67° and greater than 94° are considered slightly unappealing, and any angle exceeding the range of 67°-94° is considered very unattractive. Burnstone ^17^ stressed the importance of this angle since laypeople were more likely to evaluate upper lip protrusion in relation to the nose.

For female profiles where the upper lip E-line was decreased by 1 mm and 2 mm, and the lower lip E-line decreased by 1 mm and 2 mm, significantly more orthodontists than general dentists or laypeople felt the profiles to be unattractive as these profiles became a little convex. Similarly, for profiles of both females and males where the upper lip E-line was increased by 1 mm and 2 mm, orthodontists and general dentists gave lower scores. They felt these profiles were unattractive as they became close to class II or convex profiles. According to Young-Chel Park and Charles J Burnstone, ^18^ the upper lip E-line in the normal occlusion group is -4.8 mm, and the lower lip E-line is -3.8 mm.

Most of the study participants gave higher scores for both female and male profiles where the soft tissue pogonion (pg-pg’) was increased by 2 mm. This implied that most of them might prefer a slightly concave profile to be more esthetic. Again, for the profiles of both female and male models where the soft tissue pogonion (pg-pg’) was increased by 4 mm (as it became more class III as well as deemed unappealing) and for a profile where soft tissue pogonion (pg-pg’) was decreased by 4 mm, most of the orthodontists significantly considered these profiles unattractive. Additionally, it showed a maximum disparity in orthodontists’ perception compared to both groups. The level of training and experience among orthodontists and general dentists may differ, potentially influencing their esthetic judgments. According to Park and Burnstone, pg-pg’ was found to be 12.2 mm in the normal group and increased to 13.2 mm in class II division 1 patients; mean values for females were 12.6 mm and 13 mm for males. In the original profile of male and female study models with normal pg-pg’, all three groups felt the profiles average or good-looking. ^18^

Regarding perceptions of the groups for oral surgery, significantly more orthodontists considered the need for surgery to improve the facial esthetics for a female profile with the lower lip E-line decreased by 2 mm. Similarly, in the female profiles where the soft tissue pogonion (pg-pg’) was increased by 4 mm and decreased by 4 mm, most of the orthodontists believed these profiles needed surgery compared to other groups. It was noticed that although there was no statistically significant difference for the profiles with a decreased 2 mm from the upper lip E-line and a decreased 2 mm from the Lower lip E-line, more orthodontists preferred surgery. Perceptions of different groups for assessing the need for surgery for male profiles found that significantly more orthodontists preferred surgery for a profile where the nasolabial angle was increased by 4 degrees compared to other groups. Also, more orthodontists preferred surgery for the profiles with decreased nasolabial angle by -4° for both males and females as the profiles became more class II and convex. It was noticed that for most of the original study model profiles, and a profile with soft tissue pogonion (pg-pg’) increased by 2 mm, almost 100% percent of them felt that surgery was not required to improve the facial esthetics. DeAlmeida and Bittencourt in their study stated that surgery was indicated mainly for the convex profiles for males and concave profiles for females. ^19^

Limitations: Esthetic preferences are inherently subjective, and individual biases may affect responses, leading to variability in preferences that may not reflect a consensus. Cultural norms and values can influence esthetic preferences. The study may not account for regional or cultural differences in beauty standards, potentially limiting the applicability of the findings across diverse populations. These limitations highlight the importance of carefully interpreting and considering the results alongside other research for a more comprehensive understanding.

Recommendations: Further studies can compare findings with other cultural contexts to explore how aesthetic preferences differ globally. Incorporating qualitative methods, such as interviews or focus groups, is recommended to gain deeper insights into the reasoning behind esthetic preferences. It is also recommended to utilize 3D imaging or virtual simulations to enhance the assessment of facial profiles and allow participants to visualize changes more effectively. Implementing these recommendations could enhance the robustness and applicability of future research in esthetic preferences.

## Conclusions

Hence, it was concluded that participants in the three groups had diverse conceptions of facial attractiveness in all parameters considered. Compared to general dentists and laypersons, orthodontists were much more precise, firmer, and meticulous in identifying a favorable or good-looking profile. In the present study, orthodontists attributed lower pleasantness scores to almost all the altered female and male facial profiles. The study results also indicate that although more orthodontists identified the need for surgical correction for a few severely distorted profiles, there was a statistically non-significant difference between the groups for most of the altered profiles. Since esthetics is subjective and leads to different facial evaluations, orthodontists must address patients’ esthetic concerns for satisfactory treatment outcomes.

## Informed consent

We confirm that we have obtained permission to use images and data from the participants included in this work.

## Data Availability

Mendeley Data: Esthetic Preferences of Orthodontists, general dentists, and Laypersons for Indian facial profiles: A cross-sectional study, https://doi.org/10.17632/b9tkhwjhjb.1. ^20^ This project contains the following underlying data:
- Raw Data - Aesthetic Preferences of various study groups.xlsx Data are available under the terms of the Creative Commons Attribution 4.0 International license (CC-BY 4.0).
