# Supplementary material for: Esthetic preferences of orthodontists, general dentists, and laypersons for Indian facial profiles: A cross-sectional study
Source: F1000Res. 2024 Sep 30;12:953. Originally published 2023 Aug 8. [Version 2] doi: 10.12688/f1000research.138742.2 (PMC11456152; doi:10.12688/f1000research.138742.2)
Supplement: Peer review history

# Reviewer report: Reviewer response for version 2

**DOI:** 10.5256/f1000research.172422.r328130

**Published:** 2024-10-10

**Authors:** In Meei Tew

**Affiliations:** 1 Department of Restorative Dentistry, Universiti Kebangsaan Malaysia, Kuala Lumpur, Malaysia

The authors have addressed all the previous comments,  but I have a few additional suggestions for authors' consideration:

 i) suggest to remove the word "Limitation:" and add one sentence at the beginning of the paragraph. For example: This study has several limitations......"

 ii) suggest to remove the word "recommendations:" 

 Otherwise, this revised manuscript will be ready to be indexed.

Is the work clearly and accurately presented and does it cite the current literature?

Yes

If applicable, is the statistical analysis and its interpretation appropriate?

Yes

Are all the source data underlying the results available to ensure full reproducibility?

Yes

Is the study design appropriate and is the work technically sound?

Yes

Are the conclusions drawn adequately supported by the results?

Yes

Are sufficient details of methods and analysis provided to allow replication by others?

Yes

Reviewer Expertise:

NA

I confirm that I have read this submission and believe that I have an appropriate level of expertise to confirm that it is of an acceptable scientific standard.

# Reviewer report: Reviewer response for version 2

**DOI:** 10.5256/f1000research.172422.r328129

**Published:** 2024-10-04

**Authors:** Dinesh Rokaya

**Affiliations:** 1 Walailak University, Thai Buri, Nakhon Si Thammarat, Thailand

The authors  have corrected the article and it can be accepted.

Is the work clearly and accurately presented and does it cite the current literature?

Partly

If applicable, is the statistical analysis and its interpretation appropriate?

Yes

Are all the source data underlying the results available to ensure full reproducibility?

Partly

Is the study design appropriate and is the work technically sound?

No

Are the conclusions drawn adequately supported by the results?

Partly

Are sufficient details of methods and analysis provided to allow replication by others?

Partly

Reviewer Expertise:

Prosthodontics, Esthetic Dentistry, Biomaterials

I confirm that I have read this submission and believe that I have an appropriate level of expertise to confirm that it is of an acceptable scientific standard.

# Reviewer report: Reviewer response for version 1

**DOI:** 10.5256/f1000research.151968.r222335

**Published:** 2024-07-03

**Authors:** Pawankumar Tekale

**Affiliations:** 1 Dr. Rajesh Ramdasji Kambe Dental College and Hospital, Maharashtra, India

Overall research is nice. This study would have given more valuable conclusive inputs if the sample size could have been 25-30 in each group. Furthermore, recent references need to be added. As only a couple of reference articles between 2020 to 2024 and 3 articles between 2010 to 2020. Please try to remove citations from the conclusion and reframe the conclusion in a single paragraph with 4-5 lines only so that researchers and clinicians will be able to line up the clinical implications.

Is the work clearly and accurately presented and does it cite the current literature?

Yes

If applicable, is the statistical analysis and its interpretation appropriate?

Yes

Are all the source data underlying the results available to ensure full reproducibility?

Yes

Is the study design appropriate and is the work technically sound?

Yes

Are the conclusions drawn adequately supported by the results?

Yes

Are sufficient details of methods and analysis provided to allow replication by others?

Yes

Reviewer Expertise:

I am a researcher and clinician in orthodontics specialty

I confirm that I have read this submission and believe that I have an appropriate level of expertise to confirm that it is of an acceptable scientific standard.

# Reviewer report: Reviewer response for version 1

**DOI:** 10.5256/f1000research.151968.r242357

**Published:** 2024-02-20

**Authors:** Dinesh Rokaya

**Affiliations:** 1 Walailak University, Thai Buri, Nakhon Si Thammarat, Thailand

This is an interesting study where the researchers compared the perceptions of a group of orthodontists, general dentists, and laypersons about the attractiveness of Indian facial profiles.

 Some Comments.

 There exist differences in facial aesthetics measurements in males and females. Please refer to the article (Shah P. et. al, 2022 [ref 1]).

 Considering the objective of this study, the total sample size is only 54 (18 subjects in each group). It would have been better if they had included more samples in each group. The authors need to mention how they calculate the sample size. Which formula or software they used?

 Was the questionnaire used in this study taken from other sources or self-made? Was the questionnaire validated? Please explain.

 Add the inclusion criteria of the subjects.

 Can this study be generalized?

 If this study is conducted in larger samples or different settings, will the results be the same or changed? Please discuss.

 Add limitations of this research.

Is the work clearly and accurately presented and does it cite the current literature?

Partly

If applicable, is the statistical analysis and its interpretation appropriate?

Yes

Are all the source data underlying the results available to ensure full reproducibility?

Partly

Is the study design appropriate and is the work technically sound?

No

Are the conclusions drawn adequately supported by the results?

Partly

Are sufficient details of methods and analysis provided to allow replication by others?

Partly

Reviewer Expertise:

Prosthodontics, Esthetic Dentistry, Biomaterials

I confirm that I have read this submission and believe that I have an appropriate level of expertise to state that I do not consider it to be of an acceptable scientific standard, for reasons outlined above.

## References

[1] : Facial Esthetic Analysis of Nepalese Subjects. *The Open Dentistry Journal* .2022;16(1) : 10.2174/18742106-v16-e2111191 10.2174/18742106-v16-e2111191

# Reviewer report: Reviewer response for version 1

**DOI:** 10.5256/f1000research.151968.r209431

**Published:** 2023-10-16

**Authors:** In Meei Tew

**Affiliations:** 1 Department of Restorative Dentistry, Universiti Kebangsaan Malaysia, Kuala Lumpur, Malaysia

This is a cross-sectional study on the comparison of esthetic preferences of orthodontists, general dentists, and laypersons for Indian facial profiles. It is generally well written, but there are some comments or suggestions for authors' kind consideration:

 Abstract:

 Methods:

 There are a few sentences required modification for further clarification:

1. A visual analog scale (VAS) along with a question about surgical correction opinion was given to 18 orthodontists, 18 general dentists, and 18 laypersons to score (1-5) from least to most attractive.
2. Spearman’s rank correlation was computed to assess correlation, as well as ANOVA, followed by *post hoc *Tukey analysis to compare the mean scores, and Chi-square test to determine the opinion about surgical treatment.

Introduction:

- suggest to remove first paragraph as the contents have been well covered in second paragraph

Statistical data analysis:

- Need a further clarification on “orthodontic education” in the second sentence.
- Table 1 to Table 3: Kindly place the * behind the value which is less than 0.05 in the Table.

Discussion:

 Kindly include the study limitations and further recommendation at the last paragraph.

 Conclusions:

 Kindly provide a concise conclusion which match with the objective of the study

Is the work clearly and accurately presented and does it cite the current literature?

Yes

If applicable, is the statistical analysis and its interpretation appropriate?

Yes

Are all the source data underlying the results available to ensure full reproducibility?

Yes

Is the study design appropriate and is the work technically sound?

Yes

Are the conclusions drawn adequately supported by the results?

Yes

Are sufficient details of methods and analysis provided to allow replication by others?

Yes

Reviewer Expertise:

Prosthodontics

I confirm that I have read this submission and believe that I have an appropriate level of expertise to confirm that it is of an acceptable scientific standard, however I have significant reservations, as outlined above.

# Response

**Published:** 2024-09-24

**Authors:** Nishu Singla

**Affiliations:** Public Health Dentistry, Manipal Academy of Higher Education, Manipal, Karnataka, India

I have corrected the manuscript per **In Meei Tew's **suggestions from the Department of Restorative Dentistry, Universiti Kebangsaan Malaysia, Kuala Lumpur, Malaysia.

 I have made the following changes to the article **"Esthetic Preferences of Orthodontists, General Dentists, and Laypersons for Indian Facial Profiles: A Cross-sectional Study."**   

 Abstract: no change

 Introduction: Grammatical corrections done

 Methods: Rephrased the sentences as suggested by the reviewer. 

 Statistical data analysis: Clarification on “orthodontic education” in the second sentence.

 Table 1 to Table 3: Placed the * behind the value, which is less than 0.05, in the tables.

 Discussion: The study limitations and recommendations are now included in the last paragraph.

 Conclusions: The conclusion is corrected according to the objectives.
